# Significant burden of post-COVID exertional dyspnoea in a South-Italy region: knowledge of risk factors might prevent further critical overload on the healthcare system

**DOI:** 10.3389/fpubh.2023.1273853

**Published:** 2023-12-21

**Authors:** Emanuela Resta, Eustachio Cuscianna, Paola Pierucci, Carlo Custodero, Vincenzo Solfrizzi, Carlo Sabbà, Chiara Maria Palmisano, Federica Barratta, Maria Luisa De Candia, Maria Grazia Tummolo, Elena Capozza, Sonia Lomuscio, Lucrezia De Michele, Silvio Tafuri, Onofrio Resta, Gennaro Mariano Lenato

**Affiliations:** ^1^University of Foggia – Doctorate School of Translational Medicine and Management of Health Systems, Foggia, Italy; ^2^Dipartimento Interdisciplinare di Medicina, Università degli Studi di Bari Aldo Moro, Bari, Italy; ^3^Policlinico Hospital, University of Bari Aldo Moro – Respiratory Medicine Unit, Bari, Italy; ^4^Policlinico Hospital – University of Bari Aldo Moro – “Frugoni” Internal Medicine and Geriatric Unit, Bari, Italy; ^5^“POC Central-SS. Annunziata-Moscati” Taranto Hospital – Pulmonology Unit, Taranto, Italy; ^6^Bari "San Paolo" Hospital – Pulmonology Unit, Bari, Italy; ^7^Bari "Di Venere" Hospital – Pulmonology Unit, Bari, Italy; ^8^Terlizzi “Sarcone” Hospital – Pulmonology and Respiratory Rehabilitation Unit, Terlizzi, Italy; ^9^Department of Biomedicine and Prevention, Tor Vergata University of Rome, Rome, Italy; ^10^Policlinico Hospital – University of Bari Aldo Moro – Cardiology Unit, Bari, Italy; ^11^Policlinico Hospital – University of Bari Aldo Moro – Post-COVID Unit Service of Respiratory Medicine, Bari, Italy

**Keywords:** post-COVID syndrome, healthcare burden, healthcare capacity, post-COVID exertional dyspnoea, fatigue

## Abstract

**Background:**

Exertional dyspnoea in post-COVID syndrome is a debilitating manifestation, requiring appropriate comprehensive management. However, limited-resources healthcare systems might be unable to expand their healthcare-providing capacity and are expected to be overwhelmed by increasing healthcare demand. Furthermore, since post-COVID exertional dyspnoea is regarded to represent an umbrella term, encompassing several clinical conditions, stratification of patients with post-COVID exertional dyspnoea, depending on risk factors and underlying aetiologies might provide useful for healthcare optimization and potentially help relieve healthcare service from overload. Hence, we aimed to investigate the frequency, functional characterization, and predictors of post-COVID exertional dyspnoea in a large cohort of post-COVID patients in Apulia, Italy, at 3-month post-acute SARS-CoV-2 infection.

**Methods:**

A cohort of laboratory-confirmed 318 patients, both domiciliary or hospitalized, was evaluated in a post-COVID Unit outpatient setting. Post-COVID exertional dyspnoea and other post-COVID syndrome manifestations were collected by medical history. Functional characterization of post-COVID exertional dyspnoea was performed through a 6-min walking test (6-mwt). The association of post-COVID exertional dyspnoea with possible risk factors was investigated through univariate and multivariate logistic regression analysis.

**Results:**

At medical evaluation, post-COVID exertional dyspnoea was reported by as many as 190/318 patients (59.7%), showing relatively high prevalence also in domiciliary-course patients. However, functional characterization disclosed a 6-mwt-based desaturation walking drop in only 24.1% of instrumental post-COVID exertional dyspnoea patients. Multivariate analysis identified five independent predictors significantly contributing to PCED, namely post-COVID-fatigue, pre-existing respiratory co-morbidities, non-asthmatic allergy history, age, and acute-phase-dyspnoea. Sex-restricted multivariate analysis identified a differential risk pattern for males (pre-existing respiratory co-morbidities, age, acute-phase-dyspnoea) and females (post-COVID-fatigue and acute-phase-dyspnoea).

**Conclusion:**

Our findings revealed that post-COVID exertional dyspnoea is characterized by relevant clinical burden, with potential further strain on healthcare systems, already weakened by pandemic waves. Sex-based subgroup analysis reveals sex-specific dyspnoea-underlying risk profiles and pathogenic mechanisms. Knowledge of sex-specific risk-determining factors might help optimize personalized care management and healthcare resources.

## Introduction

Post-COVID-19 syndrome is a multisystem disease developing in patients with prior SARS-CoV-2 infection, characterized by a wide range of persistent clinical symptoms, occurring in hospitalized as well as in patients with relatively mild acute-phase illness (1–3). According to recent epidemiologic estimates, such an emerging condition is thought to affect 65–144 million individuals worldwide (4–6).

Since a notable portion of subjects affected by post-COVID syndrome reports lingering and debilitating symptoms (7, 8), such as dyspnoea and exertional intolerance (9), often associated with impairment of daily life activities (10), this new chronic health condition is expected to result in a considerable societal impact, potentially leading to economically relevant consequences, in terms of days off from work and utilization of healthcare resources and management (2, 6, 11). Appropriate management strategies specifically addressed to target post-COVID patients with clinically significant exertional dyspnoea should be established by healthcare systems and policymakers (1, 12–14). However, the limited capacity of healthcare systems would represent a paramount critical issue, in light of the significant restrictions and resource redirection from the usual chronic to acute healthcare settings, during the pandemic peaks (6, 15).

Furthermore, such a scenario is expected to become particularly challenging in those socioeconomic and/or geographic areas already facing shortages of medical equipment and care facilities. Many of Southern Italy’s regions were subjected to considerable cuts and healthcare restrictions to chronic respiratory disease management in the last decade, with consequent vulnerability to saturation of healthcare facilities (16, 17). When subjected to this additional strain, after the already-devastating pandemic waves, such healthcare systems might be led close to the risk of collapse (15–17). Knowledge of predictors for severe post-COVID syndrome-related dyspnoea might help identify high-risk patients and potentially relieve healthcare service from overload (1, 18).

However, the risk factors underlying dyspnoea associated with post-COVID syndrome are yet to be elucidated (1, 2, 19). Remarkably, an apparent lack of concordance between the presence of subjective exertional intolerance and results of pulmonary functional or radiological investigations has been observed in several studies (1, 18, 20), in that up to 35–65% of patients complained of dyspneic symptomatology despite normal pulmonary function test and chest CT imaging profile (14, 21–23). This study aimed to characterize a large cohort of post-COVID patients in Apulia, Italy, in the setting of a multi-disciplinary dedicated post-COVID Unit, to estimate the frequency of new or persistent dyspnoea in the post-acute-COVID-19-episode phase, in both hospital- and domiciliary-management patients, and to investigate predictors of post-COVID dyspnoea and reasons for lack of return to baseline health status at follow-up.

## Materials and methods

### Study design

This study was carried out in the Respiratory Post-COVID-19 Syndrome outpatient specialist service, specifically established at the Pulmonology Unit of the University Policlinico Hospital of Bari (Apulia, Italy). The Post-COVID-19 Syndrome clinical service project is an ongoing initiative developed by the University Policlinico Hospital of Bari aimed to evaluate the long-term impact of COVID-19 on the respiratory system and offer healthcare to patients (> 16 years old) residents in the Italian Apulia Region and potentially needing management for Post-COVID Exertional Dyspnoea (PCED). Service setting up included a first assessment protocol scheduled at 3 months post-acute-SARS-CoV-2-infection and a subsequent follow-up protocol after the first consultation depending on the grade of severity and persistence of symptoms. The service was available for all post-COVID patients, regardless of symptoms or acute-phase healthcare setting.

The study was designed as a retrospective cross-sectional observational survey. Patients attended the clinical service for Post-COVID Syndrome assessment throughout the pandemic period. Results of the 3-month post-acute-SARS-CoV-2-infection assessment are reported herewith (recruitment period January 2021–August 2021).

Appropriate information about the use of personal data was given to all patients cared for in the clinic and also regarded the possible use of collected data for publication. In the information, it was clarified that data will be used according to Italian law about the protection of personal data. Signed informed content was obtained from each participant.

The study was conducted in accordance with the principles of the 1964 Declaration of Helsinki.

The protocol of the study was communicated to the Puglia Observatory for Epidemiology.

### Study population

All patients had received molecular/antigen-based laboratory confirmation of SARS-CoV-2 infection by nasal/oral swab-based analysis. At the time of the evaluation, all patients had received recovery confirmation by achieving molecular/antigen SARS-CoV-2 swab test negativization.

### Data collection

The assessment protocol included medical history collecting both remote pre-COVID-19 clinical conditions and information on acute-phase-COVID-19 episodes (both respiratory and non-respiratory clinical symptoms, domiciliary/hospitalization course, hospitalization setting when applicable, need for respiratory/ventilatory support, medications). Clinical severity during the COVID-19 acute phase was classified as follows: (1) domiciliary course; (2) hospitalization in a General Medicine setting; (3) hospitalization in a Pulmonology/Semi-Intensive Care setting; (4) ICU admission. (24, 25). Careful clinical evaluation of Post-COVID-related medical history included both current respiratory (self-reported PCED, coughing, chest pain/breathing discomfort) and non-respiratory (fatigue, joint/muscle pain, gustative sensory impairment, olfactory sensory impairment, fever, cephalalgia/headache/cognitive fog, tachycardia, alopecia, anxiety/depression) clinical symptoms (9, 26, 27). Clinical symptoms were collected upon semi-structured interview-based specific questions during clinical examination, by the visiting physician. Medical evaluation of respiratory health status included signs of peripheral desaturation, resting room pulse-oximetry, and walking drop during 6mwt.

### Instrumental physical evaluation

Instrumental characterization of PCED was carried out through a 6-min-walking-test (6-mwt) and measuring distance run, completed/interrupted 6-mwt, difference pre-post 6-mwt in subjective perceived exertion using the Borg-Category-Ratio-10 (a scale ranging from 0 to 10, in which 10 represents extreme intensity of activity), and pre-post 6-mwt peripheral SatHbO_2_ walking drop. A 6-mwt test interruption and/or shorter distance run indicate worse performance. Instrumental PCED was defined as a 6-mwt-induced increase of ≥2 units in Borg-Category-Ratio-10 (DeltaBorgScale≥2) and/or 6-mwt test interruption. Instrumental PCED was also compared to self-reported PCED in medical history. To gain insights into the pathogenic mechanism underlying Post-COVID dyspnoea, instrumental PCED-suffering patients were classified according to peripheral SatHbO_2_ walking drop during 6-mwt (DeltaSatHbO_2_ ≤ −2% indicating presumably dyspnoea-underlying respiratory dysfunction, otherwise indicate presumably other dyspnoea-underlying mechanisms).

### Statistical analysis

Descriptive statistics were used to describe clinical features of the study population according to pre-existing clinical conditions, acute-phase-COVID-19 symptoms, and post-COVID-related manifestations. Continuous variables were expressed as mean ± SD. Categorical variables were expressed as frequencies by absolute value and percentage (%) of the total. Differences in the population subsets were assessed by the Mann–Whitney U-test for continuous variables and Fisher’s z-Exact Test for categorical variables. For each post-COVID syndrome-related clinical manifestation, persistence rate and new-onset rate are reported. Persistence rate is reported as a ratio between the number of patients showing each single symptom at 3-month follow-up divided by the number of patients showing the same symptom in the acute phase. New-onset rate is reported as a ratio between the number of patients showing each single symptom at 3-month follow-up divided by the number of patients lacking the same symptom in the acute phase. For each value of persistence rate and new-onset rate, 95% CI was reported, assuming a binomial distribution. Differences in persistent rate and new-onset rate for each post-COVID syndrome-related clinical manifestation were analyzed by the McNemar test.

To identify predictors of PCED, a multivariate logistic regression model was established. Acute-phase dyspnoea, PCED, acute-phase fatigue, and post-COVID fatigue were used as outcome variables of the model, respectively. Variables were included as covariates in the model according to their clinical significance and/or their significant difference in the univariate analysis (fully adjusted multivariate logistic regression model). Furthermore, for each of the predictor variables, partial multivariate logistic regression analysis was also performed by including only sex and age as covariates. Furthermore, multivariate logistic regression analysis was refined by splitting up the study cohort according to sex. All analyses were performed using SPSS software (version 23.0, SPSS Inc., Chicago, IL, United States). The statistical significance threshold was set at 0.05.

## Results

### Cohort

A total of 318 consecutive patients attended the Post-COVID-19 outpatient service (age 54.17 ± 14.63 years, range 16–89 yrs.; female sex ratio: 159/318, 50.0%). The mean period of follow-up (from disease onset) was 115.53 ± 61.466 days (Table 1). No sex-related statistically significant differences were found in age distribution (54.30 ± 14.13 vs. 54.03 ± 15.17 in males vs. females, respectively, *p* = 0.886), diagnostic pathway, acute disease duration, and follow-up length. Conversely, older patients had a significantly longer acute disease duration (*p* = 0.004) and length of follow-up (*p* = 0.033). A total of 311/318 patients (97.8%) had at least one symptom during the acute infection phase. Fever was the commonest reported symptom (233/318 patients, 73.3%), followed by fatigue and dyspnoea (215/318, 67.9%, and 198/318, 62.3%, respectively). The number of patients suffering from respiratory insufficiency was up to 216/318 (67.6%).

**Table 1:** Characteristics of post-COVID syndrome cohort.

Cohort	Hospitalized (N = 79)	Domiciliary (N = 239)	Total cohort (*N* = 318)	*p-value*
*Patients’ characteristics (n = 318)*				
Age, years (mean ± SD) [Range]	61.66 ± 12.83 [31;87]	51.67 ± 14.36 [16;89]	54.17 ± 14.63 [16;89]	0.000
Age distribution <55 yrs., # (%)	25/79 (31.6)	147/239 (61.5)	172/318 (54.1)	0.000
Female Sex, # (%)	27/79 (34.1)	132/239 (55.2)	159/318 (50.0)	0.002
Duration of Disease (Onset-to- negativization), days (mean ± SD) [Range]	35.39 ± 16.13 [4;95]	30.55 ± 12.91 [3;94]	31.78 ± 13.93 [3;95]	0.014
Length of Follow-up (from Disease Onset, days) (mean ± SD) [Range]	127.24 ± 67.136 [35–398]	111.62 ± 59.092 [14–458]	115.53 ± 61.47 [14–458]	0.061
*Clinical symptomatology (n = 318)*				
At least one Symptom, # (%)	79 (100)	234	313 (98.4)	0.337
None (Asymptomatic), # (%)	0	5	5 (1.6)	
Symptomatic before swab-test diagnosis, # (%)	76	218	294 (92.4)	0.217
Asymptomatic before swab-test diagnosis, # (%)	3	21	24 (7.5)	
Dyspnoea, # (%)	61	137	198 (62.3)	0.002
Fatigue, # (%)	57	158	215 (67.6)	0.336
Fever, # (%)	63	170	233 (73.3)	0.145
Coughing, # (%)	41	143	134 (42.1)	0.238
Dyspnoea and/or Respiratory insufficiency, # (%)	79	137	216 (67.9)	0.000

Healthcare setting in acute phase, hospitalization ward (*n* = 318)	Hospitalized	Domiciliary	Total cohort
Nr,**# (%)**	**79 (24.8)**	**239 (75.2)**	**318 (100)**
General Medicine care, # (%)	43 (13.5)		
Semi-Intensive care, # (%)	21 (6.6)		
Intensive care, # (%)	15 (4.7)		

Demographic and clinical data are reported according to the healthcare setting (Hospitalized vs. Domiciliary) during the acute infection phase.

During the acute phase, 79/318 patients (24.8%) needed hospitalization-based care and suffered from respiratory insufficiency needing hospitalization during the acute phase, whereas the remaining 239 patients displayed domiciliary management (Table 1). Hospitalized patients were significantly older (61.66 ± 12.839 vs. 51.67 ± 14.364, *p* = 0.001), had a significantly longer acute-disease duration (35.39 ± 16.13 vs. 30.55 ± 12.91 days, *p* = 0.014) and had a greater frequency of acute-phase dyspnoea (61/79, 77.2% vs. 137/239, 57.3%, *p* = 0.002) compared to domiciliary patients, respectively. Clinical manifestations in the acute phase displayed a typical sex-related pattern, with fever mostly affecting male patients and olfactory impairment, chest pain, cephalalgia, and diarrhea more frequently reported by female patients However, no sex-related statistically significant differences were found in age distribution, diagnostic pathway, acute disease duration, and follow-up length. Furthermore, male patients had an increased risk for unfavorable evolution requiring hospitalization (52/159, 32.7%, vs. 27/159, 17%, respectively, *p* = 0.002).

### Post-COVID manifestations

At least one Post-COVID Syndrome clinical manifestation at follow-up was reported by 243/318 patients (76.4%). The commonest reported feature was by far exertional dyspnoea, complained of by 190/318 patients (59.7%), followed by fatigue (96/318, 30.2%) and coughing (55/318 patients, 17.3%). At least one Post-COVID-Syndrome-related respiratory symptom was reported by 206/318 patients (64.8%). A large proportion of patients showed multiple concomitant respiratory or non-respiratory manifestations, with 141 (44.3%) individuals reporting ≥2 symptoms. Results are shown in Table 2.

**Table 2:** Clinical data of Post-COVID Syndrome cohort, at 3 months follow-up.

Clinical symptomatology. Post-COVID at 3 months follow-up (*n* = 318)	
Symptom	# (%)
At least one Post-COVID Syndrome-related Symptom	243 (76.4)
Post-COVID exertional dyspnoea (PCED)	190 (59.7)
Fatigue	96 (30.2)
Joint/Muscle Pain	40 (12.6)
Gustative sensory impairment	19 (6.0)
Olfactory sensory impairment	24 (7.5)
Fever	3 (0.9)
Cephalalgia/Headache/Cognitive Fog	19 (6.0)
Coughing	55 (17.3)
Breathing Discomfort/Chest Pain	32 (10.1)
Tachycardia	9 (2.8)
Alopecia	12 (3.8)
Anxiety/Depression	9 (2.8)
At least one Post-COVID Syndrome-related respiratory symptom	206 (64.8)
At least one Post-COVID Syndrome-related non-respiratory symptom	143 (45.0)
Multiple (≥2) Post-COVID Syndrome-related symptoms	141 (44.3)

The frequency of each manifestation reported in the Post-COVID phase is shown. PCED: Post-COVID exertional dyspnoea.

### Clinical burden of post-COVID exertional dyspnoea

The presence of PCED showed no significant relationship with clinical settings during the acute phase, as it was reported in a similar percentage in hospitalized vs. domiciliary patients (50/79, 63.3% vs. 140/239, 58.6%, respectively; *p* = 0.503). Interestingly, the frequency of exertional dyspnoea was significantly higher in Post-COVID-fatigue-suffering vs. fatigue-free patients (68/96, 70.8% vs. 122/222, 54.9%, respectively, *p* = 0.009), whereas no such increase is evident concerning patients suffering from fatigue during acute phase infection (133/215, 61.9% vs. 57/103, 55.3%, *p* = 0.274), thus suggesting that Post-COVID fatigue contributes to PCED, likely through a mechanism independent from acute-phase dyspnoea. Age turned out to be a significant risk factor for the frequency of exertional dyspnoea, which was reported significantly more often by old patients compared to young patients (*p* = 0.021). Nonetheless, the clinical burden of PCED was not specific for older adult patients only, since a notable portion (57/190, 30.0%) of PCED-reporting patients were < 50 yrs., most of which characterized by a domiciliary course (54/57, 94.7%), a scenario being consistent across patients with both persistent dyspnoea and new-onset dyspnoea.

### Persistence and new-onset rate

Although the majority of PCED-reporting patients had suffered from subjective dyspnoea during the acute infection phase as well (143/190, 75.3%) or desaturation during hospitalization (11/190, 5.8%), which were consistent with the definition of “persistent dyspnoea”, a significant proportion of them (36/190, 18.9%) was negative for subjective dyspnoea/respiratory insufficiency throughout the acute infection phase (“new-onset dyspnoea”). Therefore, when globally considered, 36/318 patients of our cohort (11.3%) displayed new-onset PCED at 3-month follow-up. The persistence rate of each post-COVID symptom was variable, with a high rate for some symptoms such as fatigue, joint/muscle pain, and coughing, as well as for exertional dyspnoea, to a very low rate for fever and gustative sensory impairment. Likewise, fatigue and joint/muscle pain, as well as exertional dyspnoea, showed a considerable new-onset rate, whereas for other symptoms new-onset rate was negligible (Table 3).

**Table 3:** Post-COVID syndrome at 3 months follow-up (*n* = 318).

Post-COVID at 3 months follow-up (*n* = 318). Persistence rate and new-onset rate						
	# (%)	Persistence Rate		New onset rate		*p*-value
At least one post-COVID syndrome-related symptom	243 (76.4)					
Post-COVID exertional dyspnoea (PCED)	190/318 (59.7)	143/198*	0.72 (0.65–0.78)	47/120*	0.39 (0.30–0.48)	0.488
		154/216**	0.71 (0.65–0.77)	36/102**	0.35 (0.26–0.45)	0.022
Fatigue	96/318 (30.2)	74/215	0.34 (0.28–0.41)	22/103	0.21 (0.14–0.31)	0.000
Joint/Muscle pain	40/318 (12.6)	29/175	0.16 (0.11–0.23)	11/143	0.08 (0.04–0.14)	0.000
Gustative sensory impairment	19/318 (6.0)	16/131	0.12 (0.07–0.19)	3/187	0.02 (0.00–0.05)	0.000
Olfactory sensory impairment	24/318 (7.5)	22/136	0.16 (0.11–0.24)	2/182	0.01 (0.00–0.04)	0.000
Fever	3/318 (0.9)	3/233	0.01 (0.00–0.04)	0/85	0.00 (0.00–0.05)	0.000
Cephalalgia/Headache/Cognitive Fog	19/318 (6.0)	13/90	0.14 (0.08–0.24)	6/228	0.05 (0.02–0.10)	0.000
Coughing	55/318 (17.3)	43/184	0.23 (0.18–0.30)	12/134	0.09 (0.05–0.15)	0.000
Breathing discomfort/Chest pain	32/318 (10.1)	10/56	0.18 (0.09–0.30)	22/262	0.08 (0.05–0.12)	0.005

Persistence rate and new-onset rate of each single manifestations. Persistence rate is reported as a ratio between number of the patients showing each single symptom at 3-month follow-up divided by number of patients showing the same symptom in the acute phase. New-Onset Rate is reported as a ratio between number of patients showing each single symptom at 3-month follow-up divided by the number of patients lacking the same symptom in the acute phase. *Compared to self-reported dyspnoea as a baseline in acute-phase manifestations (198/318 patients); **Compared to respiratory insufficiency, with or without symptomatic dyspnoea, during acute phase infection as a baseline manifestation (216/318 patients).

### Sex-related effect

Noteworthy, female patients reported Post-COVID-Syndrome-related symptoms more frequently than male patients, as only 29/159 female patients were symptom-free, compared to 46/159 male patients (*p* = 0.034). The greater predominance of Post-COVID Syndrome to affect female sex was evident in both respiratory and non-respiratory symptoms (0.013 and 0.001, respectively). Several single symptoms displayed a statistically significant increase in female vs. male patients, such as coughing (*p* = 0.003), fatigue (61/159, 38.4%, vs. 35/159, 22.0%, *p* = 0.002), and alopecia (*p* = 0.001), while other symptoms, such as exertional dyspnoea, did not reach statistical significance, albeit showing a clear trend towards higher frequency in female patients compared to male patients (103/159, 64.8% vs. 87/159, 54.7%, respectively *p* = 0.086). Interestingly, after the removal of hospitalized patients, who had an increased male-to-female ratio, the female-*vs*-male greater frequency of exertional dyspnoea reached statistical significance (85/132, 64.6%, vs. 55/107, 51.4%, respectively; *p* = 0.048).

### Functional investigation

As part of the clinical evaluation of the post-COVID-19 protocol of our outpatient service, an investigation by functional 6-mwt was carried out. Instrumental 6-mwt PCED and self-reported PCED in medical history were then compared. Data from the 6-mwt investigation were available for 175 of 318 patients (Table 4). Age and sex distribution were not significantly different between patients subjected to 6-mwt vs. patients with unavailable 6-mwt (data not shown). The mean run distance was 515.41 ± 142.36) mt. Instrumental PCED was reported by 133/175 (76.0%) patients, a greater proportion if compared to self-reported PCED-suffering patients (114/175 patients, 65.1%). Furthermore, among the 133 instrumental dyspneic patients, 18 patients did not complete the 6-mwt, due to the onset of severe exertional dyspnoea (and/or thoracic/respiratory symptoms). No significant difference in terms of either basal SpO_2_ (97.91 vs. 98.29), or post-6mwt SpO_2_ (97.23 vs. 97.69) was observed in dyspneic vs. non-dyspneic patients, whereas 6-mwt run distance was only marginal reduced in dyspneic vs. non-dyspneic patients (507.15 vs. 540.95, respectively, *p* = 0.632), although such differences approached significant threshold when patients were stratified and compared with respect to self-reported PCED. When we tried to more deeply characterize the 133 instrumental PCED-affected patients, with respect to presence of acute-phase dyspnoea/respiratory insufficiency, we found that Post-COVID dyspnoea could be classified as persistent dyspnoea in 100/133 patients (75.2%) and as new-onset dyspnoea in 33/133 (24.8%) cases, thus showing an overlapping scenario with results obtained on basis of self-reported PCED. To gain better insight into the etiology of the PCED, we investigated the intrinsic respiratory contribution to exercise intolerance. Our results disclosed that most dyspneic patients had no evident functional respiratory deficit, since only 32/133 patients (24.1%) with instrumental PCED had 6-mwt-based desaturation walking drop (DeltaSatHbO_2_ ≤ −2%). Noteworthy, post-COVID fatigue was present at a higher rate, namely 44/133 (33.3%). Accordingly, the presence of instrumental 6-mwt-based PCED was not significantly correlated with the frequency of 6-mwt-based desaturation signs (*p* = 0.84), whereas it showed a statistically significant correlation with the frequency of post-COVID fatigue (*p* = 0.02).

**Table 4:** Results of functional 6-mwt on patients with available 6mwt data (*n* = 175 patients).

Post-COVID exertional dyspnoea (PCED). Results of functional 6-mwt	
Patients with 6-mwt-Characteristics (*n* = 175)	
Sex – Female ratio, # (%)	**86 (49.1)**
Age, Mean (SD), yrs	53.28 ± 14.503
Hospitalized, # (%)	**44 (25.1)**
Self-reported Post-COVID exertional dyspnoea (PCED), # (%)	114 (65.1)
Post-COVID Fatigue, # (%)	50 (28.6)
*Instrumental results*	
Completed Test, # (%)	157
Interrupted Test, # (%)	18
Basal 6mwt HbSatO_2_ fraction, Mean (SD)	98.0 ± 1.38
Post-test 6mwt HbSatO_2_, Mean (SD)	97.3 ± 2.40
6mwt run distance, Mean (SD)	515.41 ± 142.36
Instrumental Post-COVID exertional dyspnoea (PCED), # (%)^a^	133 (76.0)
Instrumental 6-mwt-based peripheral HbSatO_2_ desaturation	32/133 (24.1%)
Post-COVID Fatigue	44/133 (33.3%)

a Instrumental exertional dyspnoea is defined as completing 6mwt with ≥2 DeltaBorg Units increase and/or 6mwt interrupted test due to onset of severe exertional dyspnoea and/or thoracic pain. Post-COVID exertional dyspnoea (PCED).

### Pre-existing co-morbidities and dyspnoea

With the aim to identify pre-existing clinical conditions as possible predictors of Post-COVID Syndrome clinical manifestations, we collected information on the remote clinical history of recruited patients (Table 5). Some of the co-morbidities showed non-overlapping distribution according to sex, namely dys-metabolic co-morbidities, smoking, and cardiovascular co-morbidities which were more frequent in male patients, whereas the presence of allergy showed a non-significant trend towards a greater prevalence in female patients.

**Table 5:** Pre-existing co-morbidities in the Post-COVID cohort.

Pre-existing condition	# (%)
Comorbidities (any)	191/242 (78.9)
Respiratory Comorbidities	57/242 (23.6)
COPD	15/242 (6.2)
Asthma	24/242 (9.9)
Cardiovascular Comorbidities	100/242 (41.3)
Dis-metabolic Comorbidities	67/242 (27.7)
Diabetes/Obesity Comorbidities	27/242 (11.2)
Smoking	120/243 (49.4)
Allergy	89/243 (36.6)
Non-Asthmatic Allergy	68/242 (27.9)
Allergy (inhalants)	42/243 (17.3)
Allergy (other)	46/243 (18.9)

Clinical manifestations related to pre-COVID infection were collected by medical history, at time of 3-month Post-COVID follow-up.

Self-reported PCED was significantly associated with pre-COVID-19 respiratory co-morbidities (*p* = 0.001) and a history of allergy (*p* = 0.039). However, despite self-reported PCED being more frequent in patients with pre-COVID-19 respiratory co-morbidities, a notable proportion of young patients (<50 yrs) had mute history for pre-COVID-19 respiratory diseases (38/46, 82.6%). No association was found with other co-morbidities or smoking. Conversely, fatigue revealed no significant association with any of the considered clinical predictors, except for the previously mentioned female sex.

### Multivariate analysis

To better identify predictors of PCED, we established a multivariate logistic regression model. As shown in Table 6, the model identified five predictor variables providing independent statistically significant contributions to the risk of PCED, namely Post-COVID fatigue, pre-existing respiratory co-morbidities, history of non-asthmatic allergy, age, and acute-phase dyspnoea. Conversely, predictors of acute-phase dyspnoea displayed a different pattern (Table 6), with only one common significant predictor with PCED (pre-existing respiratory co-morbidities), and three other independent predictors specific for acute-phase dyspnoea (pre-existing cardiovascular co-morbidities, acute-phase fatigue, and hospitalization). Despite showing several sex-related differences in univariate analysis, the female sex seems to play no significant effect in multivariate analysis, on either acute-phase dyspnoea or PCED. Therefore, we decided to refine the regression analysis according to the two sex-based subgroups.

**Table 6:** Multivariate logistic regression analysis on the whole cohort (*n* = 318 patients).

Covariate		B	OR	95%CI		*p*
**(A) Outcome: Post-COVID exertional dyspnoea (PCED)**						
Age^	*Unadjusted*	0.551	1.735	1.097	2.744	0.018*
	*Fully Adjusted*	0.812	2.251	1.092	4.640	0.028*
	*Partially Adjusted*	0.559	1.749	1.103	2.773	0.017*
Pre-existing Respiratory Comorbidities	*Unadjusted*	1.231	3.426	1.631	7.198	0.001*
	*Fully Adjusted*	1.132	3.103	1.362	7.073	0.007*
	*Partially Adjusted*	1.129	3.092	1.454	6.577	0.003*
History of non-asthmatic allergy	*Unadjusted*	0.546	1.727	0.941	3.169	0.078
	*Fully Adjusted*	0.965	2.625	1.282	5.376	0.008*
	*Partially Adjusted*	0.544	1.723	0.921	3.224	0.089
Acute-Phase Dyspnoea	*Unadjusted*	1.396	4.038	2.497	6.531	0.000*
	*Fully Adjusted*	1.443	4.233	2.207	8.120	0.000*
	*Partially Adjusted*	1.406	4.079	2.497	6.665	0.000*
Post-COVID Fatigue	*Unadjusted*	−0.688	0.502	0.301	0.839	0.009*
	*Fully Adjusted*	0.903	2.466	1.194	5.094	0.015*
	*Partially Adjusted*	0.624	1.866	1.098	3.174	0.021*
**(B) Outcome: Acute-Phase Dyspnoea**						
					
Pre-existing Respiratory Comorbidities	*Unadjusted*	0.671	1.957	1.000	3.831	0.046*
	*Fully Adjusted*	0.881	2.414	1.132	5.146	0.023*
	*Partially Adjusted*	0.574	1.776	0.898	3.511	0.046*
Pre-existing Cardiovascular Comorbidities	*Unadjusted*	0.815	2.259	1.289	3.961	0.004*
	*Fully Adjusted*	0.964	2.621	1.264	5.435	0.010*
	*Partially Adjusted*	0.821	2.272	1.213	4.255	0.010*
Domiciliary Management	*Unadjusted*	−0.925	0.396	0.221	0.711	0.002*
	*Fully Adjusted*	−0.987	0.373	0.173	0.804	0.012*
	*Partially Adjusted*	−0.907	0.404	0.219	0.745	0.004*
Acute-Phase Fatigue	*Unadjusted*	0.490	1.633	1.011	2.637	0.045*
	*Fully Adjusted*	0.726	2.066	1.122	3.806	0.020*
	*Partially Adjusted*	0.478	1.613	0.994	2.615	0.046*

**(A)**: Stepwise regression analysis was performed by using Post-COVID exertional dyspnoea (PCED) as outcome variable and the following parameters as covariates: Age ≥ 55 yrs. ^, Female Sex, Pre-existing Respiratory Comorbidities, Pre-existing Cardiovascular Comorbidities, Pre-existing Dis-metabolic Comorbidities, Smoking, History of non-asthmatic allergy, Domiciliary Management, Acute-Phase Dyspnoea, Post-COVID Fatigue.

**(B)**: Stepwise regression analysis was performed by using Acute-Phase Dyspnoea as outcome variable and the following parameters as covariates: Age ≥ 55 yrs. ^, Female Sex, Pre-existing Respiratory Comorbidities, Pre-existing Cardiovascular Comorbidities, Pre-existing Dis-metabolic Comorbidities, Smoking, History of non-asthmatic allergy, Domiciliary Management, Acute-Phase Fatigue.

Results of logistic regression analysis are reported according to unadjusted analysis (univariate), multivariate analysis for multiple confounders (Fully Adjusted) and sex- and age- adjusted multivariate analysis (Partially Adjusted). Only variables showing statistically significance are shown. ^= For the purpose of statistical analysis, cut-off for age was set up at 55 yrs, which represents the median age of our cohort. * = statistically significant.

When the analysis was restricted to female patients (Table 7), only two independent significant predictors were identified by regression analysis as contributors to PCED, namely, acute-phase dyspnoea (OR: 3.085; 95%CI: 1.056–9.012, *p* = 0.007) and Post-COVID fatigue (OR: 4.069; 95%CI: 1.458–11.354, *p* = 0.039). On the other hand, when the same analysis was restricted to male patients (Table 7), the regression analysis disclosed four independent significant predictors, namely acute-phase dyspnoea, pre-existing respiratory co-morbidities, age, and history of non-asthmatic allergy. The predictive effect of history of non-asthmatic allergy on PCED in male patients is not completely clear, since it displays a statistically significant contribution in the multivariate analysis, while falling below the statistical threshold in univariate analysis. Noteworthy, sex-based subgroup analysis reveals that post-COVID fatigue represents a female-specific risk factor for PCED. Accordingly, when we set up a similar logistic regression model with Post-COVID Fatigue as the outcome variable, the sex female arose as an independent statistically significant variable (OR: 2.169; 95%CI: 1.162–4.048; *p* = 0.015), by univariate analysis (not shown). Hence, these results suggest that PCED seems to display a different pattern of underlying risk factors in female and male patients.

**Table 7:** Multivariate logistic regression analysis after splitting up the cohort according to sex.

Covariate		B	OR	95%CI		*p*
**(A) Outcome: Post-COVID exertional dyspnoea - Female only (n = 159)**						
Acute-Phase Dyspnoea	*Unadjusted*	1.521	4.577	2.281	9.185	0.000*
	*Fully Adjusted*	1.403	4.069	1.458	11.354	0.007*
	*Partially Adjusted*	1.556	4.738	2.347	9.565	0.000*
Post-COVID Fatigue	*Unadjusted*	0.923	2.518	1.227	5.165	0.012*
	*Fully Adjusted*	1.126	3.085	1.056	9.012	0.039*
	*Partially Adjusted*	0.893	2.443	1.186	5.030	0.015*
**(B) Outcome: Post-COVID exertional dyspnoea - Male only (n = 159)**						
Age^	*Unadjusted*	0.926	2.525	1.320	4.833	0.005*
	*Fully Adjusted*	1.088	2.969	1.062	8.303	0.038*
	*Partially Adjusted*	0.834	2.304	1.090	4.867	0.029*
Pre-existing Respiratory Comorbidities	*Unadjusted*	1.487	4.423	1.560	12.540	0.005*
	*Fully Adjusted*	1.504	4.499	1.357	14.921	0.014*
	*Partially Adjusted*	1.365	3.915	1.358	11.285	0.012*
History of non-asthmatic allergy	*Unadjusted*	0.774	2.168	0.909	5.170	0.081
	*Fully Adjusted*	1.189	3.283	1.176	9.166	0.023*
	*Partially Adjusted*	0.953	2.595	1.045	6.444	0.040*
Acute-Phase Dyspnoea	*Unadjusted*	1.312	3.714	1.891	7.297	0.000*
	*Fully Adjusted*	1.456	4.289	1.755	10.479	0.001*
	*Partially Adjusted*	1.296	3.654	1.821	7.330	0.000*

In both models, stepwise regression analysis was performed by using Post-COVID exertional dyspnoea (PCED) as outcome variable and the following parameters as covariates: Age ≥ 55 yrs. ^, Pre-existing Respiratory Comorbidities, Pre-existing Cardiovascular Comorbidities, Pre-existing Dis-metabolic Comorbidities, Smoking, History of non-asthmatic allergy, Domiciliary Management, Acute-Phase Dyspnoea, Post-COVID Fatigue. **(A)**: Female only. **(B)**: Male only. Results of logistic regression analysis are reported according to unadjusted analysis (univariate), multivariate analysis for multiple confounders (Fully Adjusted) and age-adjusted multivariate analysis (Partially Adjusted). Only variables showing statistically significance are shown. ^= For the purpose of statistical analysis, cut-off for age was set up at 55 yrs, which represents the median age of our cohort. * = statistically significant.

## Discussion

In the present study, we report a snapshot of the clinical features of a large cohort of patients referring to an outpatient service, specifically established for follow-up of Post-COVID Syndrome manifestations. At 3-month follow-up, patients displayed a wide range of clinical features, spanning from mild symptoms with little clinical relevance, or complete recovery, to the detection of long-term symptoms of considerable clinical significance. Our results evidenced that Post-COVID Syndrome, at 3-month post-infection remission, actually involved at least one reported clinical manifestation in a conspicuous portion of patients, with respiratory symptoms playing a pivotal role, as exertional dyspnoea revealed to be the commonest reported feature. PCED is a debilitating condition requiring targeted management, including frequent follow-up consultations and other-than-respiratory specialist evaluation (4, 12, 28, 29). A proper ongoing outpatient service is then needed in the appropriate healthcare setting, in the framework of a holistic multi-disciplinary approach, including pulmonary functional investigations, respiratory rehabilitation facilities (1, 15), and invasive and non-invasive imaging examinations (30–35). Such dedicated service would mitigate the post-COVID disease trajectory, thus potentially preventing the worsening of daily life and/or professional impairment, as well as avoiding future hospitalizations (15, 34).

Due to the high variability of PCED, comprehensive management cannot be achieved according to a “one-size-fits-all care” model (36). Rather, optimal care organization requires a personalized fashion, thus addressing different dyspnoea manifestations, severity degrees, and eventual co-morbid conditions (4, 6, 15). In this framework, it could be reasonable to think that PCED mainly develops in people with older age, with pre-existing respiratory conditions, or with acute-phase hospitalization courses (37–39). Although self-reported PCED was more frequent in patients with pre-COVID-19 respiratory co-morbidities in this cohort, our results suggest that a notable portion (57/190, 30.0%) of PCED-reporting patients were relatively young (under 50 yrs), most of whom were never admitted but remained at home and had an unremarkable medical history for pre-COVID-19 respiratory diseases (82.6%). Post-COVID multi-professional service should then sustain growing healthcare needs posed by different patients’ subsets, ranging from individuals with pre-existing respiratory conditions (whose potentially already altered lung parenchyma likely contributed to worsening acute-phase lung inflammation and consequent persistence of the exertional dyspnoea in post-COVID phase (40, 41)), to a considerable amount of relatively young and healthy dyspnoea-affected patients, who are expected to ask for increasing demand for adequate medical attention. Given their long life expectancy, the importance of ensuring adequate ongoing long-term outpatient post-COVID assistance in the latter patients’ subgroup is crucial (42, 43). Policymakers need to be aware that adequate supplies are required to potentiate healthcare delivery for PCED patients. However, expansion of healthcare capacity may be hard to achieve in those areas with resource limitations (44). In the last decade, the Apulia Region, Southern Italy, was subjected to diminished healthcare provision, reorganization of bed allocation, limitation of medical/healthcare personnel units, and reduction of respiratory rehabilitation infrastructures, with pandemic waves imparting further unprecedented strain, with consequent impaired capacity to sustain healthcare overload (16, 17).

As underlined in the results, an acute-phase hospitalization course is not a significant predictor of PCED. Furthermore, the persistence of acute-phase dyspnoea cannot fully account for the presence of PCED, since a notable portion of Post-COVID dyspneic subjects (19%) reported new-onset exertional dyspnoea, despite unremarkable acute-phase respiratory history. Our findings provide evidence that PCED is a heterogeneous nosological entity, with likely multiple underlying aetiologies. In the present study, instrumental characterization of respiratory function, based on 6-mwt, disclosed a high prevalence of PCED, even higher than subjectively reported dyspnoea, potentially due to either a higher sensitivity of instrumental 6-mwt-based approach or patients’ under-reporting during the medical interview. Noteworthy, we did not detect any statistically significant correlation between Post-COVID instrumental 6-mwt-based PCED and signs of actual pulmonary dysfunction, in terms of desaturation signs (DeltaSatHbO_2_ ≤ −2%), thus indicating that PCED should not be regarded as a condition mainly involving lung damage or impairment. Rather, we found a statistically significant correlation between instrumental 6-mwt-based PCED and the frequency of post-COVID fatigue. Several studies also suggest that PCED is a wide-range disease, potentially encompassing several potential underlying conditions (6, 19, 43). Accordingly, at least two different phenotypes/mechanisms underlying PCED arise from our results. In a portion of individuals, PCED may represent a sequela associated with marked functional pulmonary involvement/damage and manifesting in the post-COVID phase as a walking drop in peripheral oxygenation. Such respiratory impairment might be primarily explained as chronic lung damage or, alternatively, as dysregulated inflammatory cytokine response in chest respiratory muscles or persistent pulmonary microvascular thrombosis and altered alveolar diffusion (37, 38, 42). Another subset of patients in our study had no detectable peripheral desaturation, thus clearly suggesting a different pathogenic mechanism. Accordingly, a number of recent reports described PCED as a consequence of decreased peripheral oxygen delivery or muscular consumption/deconditioning, in the absence of oxygen supply limitations (1, 20, 21). Several underlying mechanisms have been evoked, such as systemic microclotting/thromboinflammation, reduced metabolic oxidative capacity, virus-induced alterations in muscle tissue, and inactivity-induced muscle loss (18, 36, 45–49). Previous studies showed that a substantial proportion of Post-COVID Syndrome-affected patients with exertional dyspnea display radiological evidence of pulmonary interstitial disease, with heterogeneous aetiologies, such as pulmonary fibrosis conditions induced by the initial COVID-19 episode, previously identified interstitial fibrosis showing SARS-CoV-2-associated worsening/deterioration, as well as previously undiagnosed interstitial fibrosis unraveled by SARS-CoV-2 infection (50, 51). Furthermore, persistent interstitial disease and pulmonary embolism-related sequelae are also part of the dyspnea-associated spectrum post-COVID-related disease (33, 52, 53). It can be surmised that a fraction of dyspneic patients and 6-mwt-associated desaturation events might have been secondary to signs of a residual thin scattered area of the lung parenchyma involved by interstitial disease, although we could not test such an assumption since the radiological thoracic examination was not routinely part of our clinical protocol and data of chronic lung affection was not collected systematically in our cohort. Indeed, despite some chest CT abnormalities (ground-glass opacities, reticulations, interstitial thickening, fibrosis, and bronchiectasis) may persist 3 months after SARS-CoV-2 infection, only a weak correlation has been reported between abnormalities observed on “resting” investigations, such as imaging, and post-COVID exertional dyspnea (51–54).

Previous studies investigating risk factors behind PCED showed conflicting results (19, 55). In the present study, we set up a multivariate regression analysis, which disclosed a quite different risk profile between acute-phase dyspnoea and exertional dyspnoea in the post-COVID phase. Post-COVID fatigue, pre-existing respiratory co-morbidities, history of non-asthmatic allergy, age, and acute-phase dyspnoea emerged as independent statistically significant contributors to PCED, whereas hospitalization did not seem to play a significant role and only represented a risk factor for acute-phase dyspnoea, thus confirming findings of univariate analysis. Similarly, a clinical history of pre-existing cardiovascular co-morbidities represents a determinant for acute-phase dyspnoea, not for PCED, since it is well known that systemic hyper-inflammatory dysregulation in COVID acute phase is mainly responsible for rapid clinical deterioration in heart-disease patients, whereas it can be surmised that such condition will not play a major role in Post-COVID phase, characterized by a likely resolution of pro-inflammatory imbalance. In order to better characterize the risk factor profile, we also set up a sex-specific analysis. Our results showed a typical sex-oriented clinical profile in COVID, with increased risk of severe disease and hospitalization risk displayed by males during the acute phase, and greater frequency of long-lasting complaints occurring in females during the Post-COVID phase, in striking accordance with the literature (56–59). Sex-restricted multivariate regression analysis reveals a partially different risk profile underlying PCED, with post-COVID fatigue representing a female-specific risk factor, whereas pre-existing respiratory co-morbidities, age, and history of non-asthmatic allergy arose as male-specific determinants. Hence, considering the two above-described different aetiologies for PCED, the pulmonary-driven mechanism seems to display a male-to-female predominance. On the opposite, fatigue-driven PCED seems to be the predominant scenario in female patients, in both univariate and multivariate analysis. In accordance, fatigue is a manifestation that is typically more frequent in female patients, both hospitalized and at home, as shown in our study and previous studies (56). Female-to-male fatigue-driven increased risk of PCED seems to involve a relatively low pulmonary impairment, but likely represents a hallmark of Post-COVID Syndrome. A potential explanation for this female increased predominance finding could be related to potential hormonal influence (57–60). Based on the results reported in the present study, which disclosed the sex-specific independent predictors underlying dyspneic symptoms, we advocate that an optimization strategy should be deployed for personalized Post-COVID Syndrome exertional dyspnoea follow-up, according to a sex-specific protocol. In primary care settings for male patients with post-COVID dyspnoea, careful attention is needed for the presence of previous respiratory conditions, which should then prompt a higher priority toward specialty-care pulmonology management. In second-level pulmonary care, an appropriate work-up should include more invasive radiological and functional thoracic surveillance for these patients’ subgroups, in light of the male-specific greater risk of worsening previous respiratory diseases. On the other hand, since Post-COVID fatigue emerged as a major contributor to PCED in female patients, particular concern on this symptom is warranted by primary care physicians, aimed to activate higher priority scores for rapid evaluation in specialty-care settings, which would then be mainly focused on thorough investigation of fatigue in a female-specific protocol. In this framework, a comprehensive assessment of fatigue by a multi-professional healthcare team should consider functional muscle-weakness investigation, neuropsychological management, and non-invasive cardiopulmonary exercise testing, aside from standard pulmonology care.

Finally, our results are based on a cohort of patients infected during the first pandemic peaks characterized by the dominance of Alpha or Delta SARS-CoV-2 variants, whereas most recent pandemic waves (with the Omicron variant and its sublineages becoming dominant) displayed attenuated acute illness and mortality but increased spreading rate (61, 62). Despite more recent SARS-CoV-2 variants seeming to be associated with a reduced odd risk of post-COVID sequelae (63) compared to earlier variants, the high absolute numbers of infected people are expected to impose a non-negligible clinical burden and a considerable concern on healthcare organizations (64). Future studies are needed to assess the degree of overlap between the risk profiles for post-COVID in the different SARS-CoV-2 variants. Interestingly, a very recent inquiry highlighted female gender as the main post-COVID-underlying risk determinant independent of the viral strain (65), which would support a strategy based on sex-specific work-up protocol as a promising approach, not only for post-COVID management of people infected by initial-SARS-CoV-2 variants, but also for individuals suffering from long-term sequelae of more recent, or currently emerging, SARS-CoV-2 variants.

Our study has some limitations. Clinical data from the acute infection phase were retrospectively collected through medical history, which could lead to recall bias. Although the post-COVID service was available for all Post-COVID-Syndrome patients, regardless of symptoms or acute-phase healthcare setting, we cannot exclude potential referral bias towards dyspnea-affected patients as expected in a pulmonology clinical setting. Instrumental data are only based on 6-mwt technique, which mirrors an overall functional capacity and may potentially be affected by several confounders and was performed in a cross-sectional manner, thus resulting in an inevitably lacking pre-COVID assessment. It was not possible to systematically collect radiologic examinations in the recruited patients, which prevented us from more characterization of different phenotypic PCED clusters. Hence, most results are based on subjective patients’ reported outcomes, rather than objective assessment, since the observational nature of the study prevented us from carrying out a deeper investigation on metabolic aerobic fitness with a mainly experimental technique such as cardiopulmonary exercise testing.

## Conclusion

Post-COVID exertional dyspnoea was revealed to be the commonest reported feature, potentially associated with a relevant clinical burden. A proper management strategy needs to be established by healthcare system institutions and policymakers, to sustain requirements for healthcare delivery and mitigate evolution towards chronicity. However, such growing healthcare demand will probably overload the insufficient capacity of the public health service in Italy and the Apulia Region, previously weakened by resource limitations and pandemic bursts. Knowledge of sex-specific risk-determining factors might help optimize personalized care management and healthcare resources.

## Data availability statement

The raw data supporting the conclusions of this article will be made available by the authors, without undue reservation.

## Ethics statement

The studies involving humans were approved by Puglia Observatory for Epidemiology. The studies were conducted in accordance with the local legislation and institutional requirements. Written informed consent for participation was not required from the participants or the participants' legal guardians/next of kin in accordance with the national legislation and institutional requirements.

## Author contributions

ER: Conceptualization, Investigation, Writing – original draft. ECu: Conceptualization, Data curation, Methodology, Writing – original draft. PP: Data curation, Methodology, Validation, Writing – review & editing. CC: Conceptualization, Data curation, Investigation, Writing – review & editing. VS: Conceptualization, Data curation, Writing – review & editing. CS: Conceptualization, Methodology, Writing – review & editing. CP: Data curation, Formal analysis, Methodology, Writing – review & editing. FB: Data curation, Methodology, Writing – review & editing. MC: Data curation, Methodology, Writing – review & editing. MT: Data curation, Investigation, Methodology, Writing – review & editing. ECa: Data curation, Investigation, Methodology, Writing – review & editing. SL: Data curation, Investigation, Methodology, Writing – review & editing. LM: Data curation, Investigation, Supervision, Writing – review & editing. ST: Conceptualization, Supervision, Writing – original draft. OR: Conceptualization, Methodology, Writing – review & editing. GL: Conceptualization, Data curation, Formal analysis, Project administration, Writing – review & editing, Writing – original draft.
